# Proteogenomics in Aid of Host–Pathogen Interaction Studies: A Bacterial Perspective

**DOI:** 10.3390/proteomes5040026

**Published:** 2017-10-11

**Authors:** Ursula Fels, Kris Gevaert, Petra Van Damme

**Affiliations:** 1 VIB-UGent Center for Medical Biotechnology, Albert Baertsoenkaai 3, B-9000 Ghent, Belgium; ursula.fels@vib-ugent.be (U.F.); kris.gevaert@vib-ugent.be (K.G.); 2 Department of Biochemistry, Ghent University, B-9000 Ghent, Belgium

**Keywords:** host–pathogen interaction, bacterial pathogens, infection biology, proteogenomics

## Abstract

By providing useful tools to study host–pathogen interactions, next-generation omics has recently enabled the study of gene expression changes in both pathogen and infected host simultaneously. However, since great discriminative power is required to study pathogen and host simultaneously throughout the infection process, the depth of quantitative gene expression profiling has proven to be unsatisfactory when focusing on bacterial pathogens, thus preferentially requiring specific strategies or the development of novel methodologies based on complementary omics approaches. In this review, we focus on the difficulties encountered when making use of proteogenomics approaches to study bacterial pathogenesis. In addition, we review different omics strategies (i.e., transcriptomics, proteomics and secretomics) and their applications for studying interactions of pathogens with their host.

## 1. General Introduction

Over the years, efforts have been made to classify microorganisms as either pathogenic or non-pathogenic, and to understand the virulence of the former. At the very beginning, virulence was thought to be an intrinsic capacity of only certain microorganisms [1]. While such a strict division is embodied in Koch postulates [2], widely used in medical microbiology since 1880, advances in the research field of infectious diseases led to a progressive flexibility of the threshold to classify a microorganism as pathogenic or not (reviewed by Pierre-Olivier Méthot and co-workers [1]). Accumulating evidence suggests the involvement of host innate and adaptive immune responses and environmental conditions as key factors in determining a pathogen’s ability to infect a host. Altogether this brings us to the current definition of virulence as ‘the outcome of a host–pathogen interaction’, thus not considering intrinsic properties of the microbe or host separately [3]. Despite significant efforts and the knowledge gained when studying microorganisms as separate entities, one of the remaining challenges of the host–pathogen interaction research field is to understand the behavior of pathogens in the context of their hosts and vice versa. Overall, this stresses the need for integrative studies of host–pathogen interactions.

More specifically, unravelling host–pathogen interactions is of central importance to understand the host response to infection and the mechanisms employed by microorganisms to subvert host defenses, and for the discovery of novel antimicrobial targets. Omics approaches have the advantage of enabling integrative studies of system alterations at different levels, including transcription, protein synthesis and alterations of the proteome amongst others. Therefore, the applications of omics-based strategies are key for the discovery of mechanisms steering bacterial virulence and pathogenesis. In this article, we focus on proteogenomics approaches, an area of research at the intersection of genomics, transcriptomics and proteomics. However, despite major advancements, proteogenomics approaches still have significant limitations when applied to study pathogens in the context of the host—a shortcoming that is mainly due to the macromolecular content of eukaryotic cells differing by several orders of magnitude from that of prokaryotes, making such studies extremely challenging. Furthermore, the lengthy procedures frequently applied to enrich for bacteria might influence the data outcome. Another limitation comes from the fact that reproduction and interpretation of reported findings is often complicated or even precluded due to the scarcity of details provided regarding the experimental setups used (Table S1). Moreover, the high variability of the infection capacity of the pathogen and the overall infection efficiency also contribute to the observed high variability in infected cell (sub-) populations. Thus, ideally, host–pathogen interactions are studied on the single cell level; however, such studies are not yet routinely performed. In this review, we focus on the application of various omics approaches that permit the study of host–pathogen interactions using bacterial pathogens as infective agents and specifically focus on studies enabling detection of proteogenomics changes in the bacterial pathogen within infected cells while not focusing on studies reporting host cell changes, since omics analysis of the latter can essentially be performed using routine procedures. For proteomic tools enabling the study of bacterial pathogen protein/host protein interactions, we would like to refer the reader to a recent review discussing such studies in more detail [4].

## 2. Current Limitations for the Application of Omics in Host–Pathogen Interaction Studies

**Figure 1:**
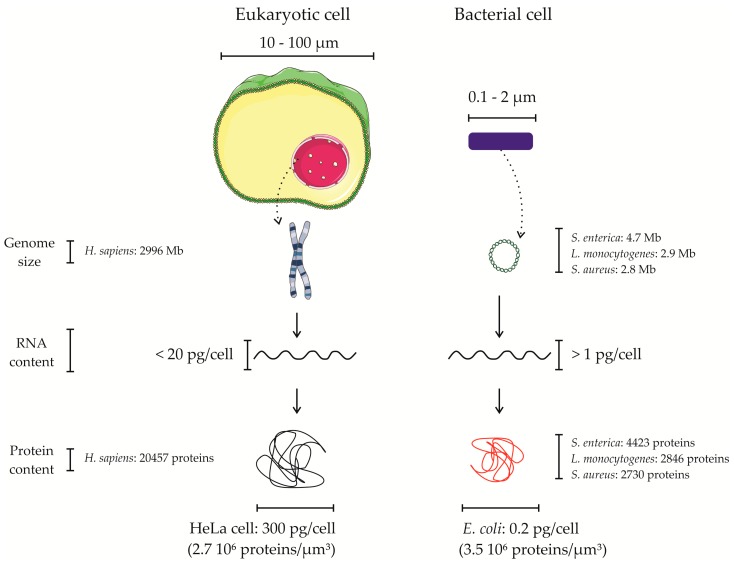
Macromolecular differences between cells of eukaryotic and bacterial origin. Eukaryotic and bacterial cells differ greatly in many aspects. First, in terms of size, these organisms are considerably different. Eukaryotic cells have a size that ranges between 10 and 100 µm, whereas bacteria are typically 0.1 to 2 µm of length. Macromolecules are the building blocks of life, present in all known living organisms. Eukaryotic cells and bacteria also have great differences in terms of their macromolecule content. Genomes differ in aspects such as size and organization, whereas the RNA content is about 20 times higher in eukaryotic cells. Finally, at the protein level, these organisms display differences not only in absolute numbers of proteins [20] and proteoforms expressed, but also in terms of the total amount of protein molecules per cell.

When applying omics approaches for studying host–pathogen interactions, it is important to consider the differences concerning the host and pathogen molecular contents (Figure 1). If the focus is on the host, studies can largely neglect the pathogen content [5,6,7]. Nonetheless, if the focus is on the pathogen’s response, its molecular content becomes limiting, making it highly desirable or even required to implement strategies to enrich the molecular content of the pathogen, strategies which are, however, not always available or easy to implement. As such, a frequently used alternative approach to study pathogenic responses is the in vitro growth of the pathogen in conditions mimicking the host environment [8,9,10,11]. In this context, it is noteworthy that despite the reliable and often ease of use of in vitro or in cellulo models to study bacterial pathogen interactions with host cells, it has become apparent that these interaction models have shortcomings in that they do not always accurately reflect in vivo infection conditions (i.e., in case of infection the lack of an immune response or difference in physiological conditions [12], bacterial micro colony formation and host cell polarity [13] amongst others) and therefore are sometimes referred to as being a reductionist approach [14]. To avoid studying cells in isolation and overcoming some of these shortcomings, in vivo models making use of a broad variety of animals ranging from invertebrates to non-human primates can be opted for depending on the bacterial pathogen of study. Sometimes however, no appropriate in vivo models are available because of the fact that the manifestation of the disease is very different from the corresponding human disease. In case of the latter, one could turn to the use of so-called ex vivo models that comprise infection of isolated tissues. For example, blood, saliva and placenta explants can be used for studying *Neisseria meningitidis* [15], *Streptococcus pyogenes* [16], and *Listeria monocytogenes* [17] infections, respectively.

To address the differences between the host and bacterial pathogen, one of the first things to consider is their difference in size. The diameter of eukaryotic cells typically ranges from 10 to 100 µm, whereas that of bacteria ranges from 0.1 to 2 µm [18]. Consequently, when considering cells as spheres, their volumes are ~10^5^ to ~10^9^ µm^3^, for bacteria and eukaryotic cells respectively. In terms of genomic content, eukaryotes possess larger and more complex genomes than prokaryotes; the genome of bacterial pathogens varies between 0.8 and 8 Mb, whereas the genome of higher eukaryotic organisms such as human (*Homo sapiens*) and mouse (*Mus musculus*) is about 2996 and 2671 Mb, respectively [19], thus about several hundred times larger. A bacterial pathogen contains approximatively 5 to 10 times less genes than their eukaryotic hosts. For example, *H. sapiens* encodes about 20,457 protein-coding genes, while *Salmonella enterica* subsp. *enterica* serovar Typhimurium (hereinafter referred to as *S. typhimurium*) strain LT2 encodes 4423 protein-coding genes [20]. As such, without considering all possible proteoforms (e.g., variant of proteins raised upon alternative splicing and alternative translation initiation amongst others) [21], eukaryotic proteomes are about one order of magnitude larger than their prokaryotic counterparts in absolute numbers of unique proteins (Figure 1).

**Figure 2:**
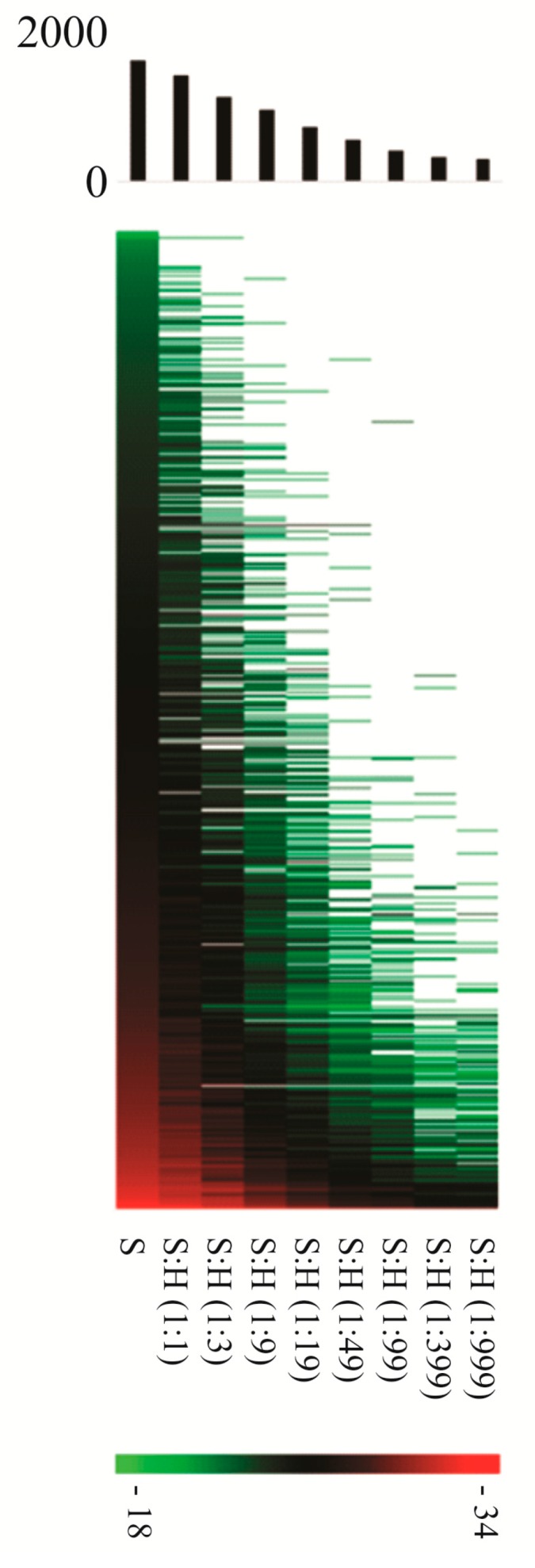
Heat map representation of identified *Salmonella* proteins and corresponding label-free quantification (LFQ) intensities when analyzing complex host/pathogen peptide mixtures. Complex peptide mixtures obtained from a trypsin digested total *Salmonella* protein lysate (S) dilution series (as indicated in the Figure) in protein lysates of human HeLa cells (H) were analyzed by liquid chromatography coupled with tandem mass spectrometry (LC-MS/MS) and the protein abundance quantified using label-free quantification (LFQ) [24]. In the heat map, the red and green color indicates high and low LFQ intensities (log2 (LFQ)) as shown by the color scale bar in log2 LFQ), respectively and white indicates that the protein was not identified in the corresponding experimental setup analyzed. In the upper part of the figure, the bar chart shows the number of proteins identified in every condition. The precise number of *Salmonella* proteins identified was 1600 (S), 1391 (S:H 1:1), 1120 (S:H 1:3), 950 (S:H 1:9), 736 (S:H 1:19), 553 (S:H 1:49), 427 (S:H 1:99), 328 (S:H 1:399), 295 (S:H 1:999).

Moreover, when studying the proteome of an infected host, the mass spectrometry-based identification of bacterial proteins is highly challenging given their overall much lower levels and the fact that despite recent improvements in accuracy, sensitivity and speed of mass spectrometers and the performance of liquid chromatography, ionization suppression and an overall low loading capacity still result in under-sampling [22]. This limits the coverage of complex samples, which can only be partially overcome by increasing the overall time spent on analyzing samples [23]. A representative example of how the number of *Salmonella* proteins identified using liquid chromatography coupled with tandem mass spectrometry (LC-MS/MS) decreases when analyzing complex peptide mixtures, being a *Salmonella* total protein dilution series in lysates of human HeLa cells, is shown in Figure 2. Here, the number of identified *Salmonella* proteins when comparing a pure *Salmonella* versus a 1:999 pathogen/host peptide mixture drops already ~5 fold (i.e., from 1600 to 295 protein identifications).

Besides the increased genome complexity of most eukaryotes, the DNA of eukaryotic cells is also organized differently from that of prokaryotic cells. Eukaryotic genetic material is organized as a macromolecular structure of highly packed DNA and proteins known as chromatin. Bacteria usually possess a unique circular chromosome and, in some cases, extrachromosomal replicons known as plasmids, which, for several bacteria such as *Shigella* species (spp.), are relevant for their virulence [25]. The RNA content of eukaryotic and prokaryotic organisms is typically about two orders of magnitude different, with about 20 pg and 0.1 pg of total RNA per eukaryotic and prokaryotic cell, respectively [26]. When considering their average sizes, the protein content of eukaryotic and prokaryotic organisms differs typically over ~1000 fold (i.e., 300 pg and 0.2 pg in case of a HeLa and *Escherichia coli* cell, respectively) [18] (Figure 1). More specifically, when considering the number of protein molecules per individual cell, a HeLa cell has been reported to contain 10^10^ proteins and an *E. coli* cell 3 × 10^6^ proteins, thus about 3000 times more proteins molecules [27]. With estimated volumes of 3000 µm^3^ and 1 µm^3^ in case of a human and *E. coli* cell, respectively, this difference is largely explained by the increase in the volume and not to an increase of protein mass per unit volume. More specifically, the protein mass per unit volume was calculated to be 2.7 × 10^6^ proteins/µm^3^ and 3.5 × 10^6^ proteins/µm^3^, for HeLa and *E. coli* cells, respectively. Altogether, this highlights that when applying omics to understand pathogenic processes involved in host–pathogen interactions, these substantial differences in molecular content cannot be neglected.

Another level of complexity is imposed by the heterogeneity among infected cells, and further, not all cells will be infected in a cell culture. More specifically, the number of bacteria per infected cell and stage of infection can (greatly) vary among infected cells (Table S1). As such, when setting up an infection model, several considerations have to be made. Some of these are the multiplicity of infection (MOI) and internalization conditions in the case of intracellular bacteria, host cell-type dependency of intracellular pathogen replication in infected cells, and viability of the infected cells and of the bacteria. Illustrative of this, upon infection of the host, *Salmonella* spp. colonize the gut, where it promotes its own internalization in epithelial cells [28]. After passing through the epithelial barrier, *S. typhimurium* can be phagocytized by macrophages and dendritic cells (DCs), explaining the widespread use of epithelial, macrophage and dendritic cell lines next to primary cell cultures (Table S1). Since a bacterial pathogen typically targets only (a) particular cell type(s) during infection, it is thus extremely important to carefully consider which representative infection model to use. As illustrated in Table S1, in the case of *Francisella tularensis*, many studies used myeloid primary cells or cell lines since these are the primary target cells and replicative niche of this bacterial pathogen. Many other different cell lines can be used as host models such as epithelial cell lines (HeLa or Caco-2), phagocytic cell lines (J774A.1 murine macrophages, THP-1 human monocytes) or primary cells such as monocyte, bone marrow derived macrophages or dendritic cells from human or murine origin, as well as neutrophils.

Most studies on intracellular pathogens use antibiotics to kill extracellular bacteria following their addition to host cell cultures enabling the focus on internalized bacteria. Generally, host cells are first seeded after which bacteria are added to the culture medium, which is followed by an incubation with the host cells for a given period (typically ranging from 30 to 60 min) to allow bacterial internalization. Then, non-internalized bacteria are washed out, and antibiotics are added to ensure killing of extracellular bacteria that adhered to the cells but were not internalized. This procedure is referred to as an antibiotic protection assay and most commonly the antibiotic gentamicin is used. With short incubation times, gentamicin does not enter host cells, hence it does not affect intracellular bacteria [29]. Following an antibiotic protection assay, intracellular bacteria are quantified by colony forming unit (CFU) enumeration or, alternatively, the number of infected cells can be estimated following fluorescence microscopy or using flow cytometry. Using microscopy, the number and the distribution or location of bacteria within host cells or cell-associated bacteria can be determined in a quantitative manner as, for example, shown by Malik-Kale et al. [30] who made use of mCherry expressing *Salmonella*. By measuring mean fluorescence intensities, flow cytometry enables semi-quantitative analyses as demonstrated by Raybourne et al. [31] who compared the intracellular *L. monocytogenes* content of human monocytes and granulocytes by pre-labeling bacteria with a lipophilic fluorescent dye. Isolation of infected cells using fluorescence-activated cell sorting (FACS) typically relies on the exogenous expression of fluorescent proteins, like green fluorescent protein (GFP), by pathogens [32,33,34,35]. Not surprisingly, several of these techniques are often used in parallel to answer complex biological questions. Moreover, it is important to highlight that expression of exogenous fluorescent proteins from plasmids can affect the virulence of *Salmonella* spp. [36]. Evidence for this was provided by Clark et al. [37] who observed a reduction in the expression of *Salmonella* pathogenic island 1 (SPI-1) genes and a reduced infectivity of bacteria containing a GFP-expressing plasmid or empty control plasmid. On the other hand, to avoid the expression of GFP protein from plasmids, the *gfp* gene can be directly cloned in the bacterial chromosome allowing for its expression from a constitutive promoter [38]. 

Another important parameter to consider is the multiplicity of infection (MOI), in bacteriology defined as the ratio of bacteria to eukaryotic host cell. This parameter should be newly established for different pathogens in relation to their hosts and their cellular states. For instance, when *Y. pestis* is used to infect macrophages, the latter may undergo apoptosis [39], which shows the importance of the appropriate MOI selection dependent of the research question. However, often it is not easy to find a consensus MOI between different studies (Table S1) as, for example, *L. monocytogenes* internalization was shown to be significantly different when comparing MOIs of 1 and 10 amongst different studies [40]. Furthermore, using the same approach, it was reported that the percentage of infected cells at 4 and 24 hpi depends on the MOI used to infect cells [38]. The authors reported that more than 75% of HeLa cells infected with *S. typhimurium* are infected at 4 hpi when an MOI of 100 is used, whereas an MOI of 10 only resulted in 5% of the cells being infected. As such, caution is needed when extrapolating the results from different studies. Generally, increasing the MOI will increase the percentage of infected cells, as shown when infecting endothelial cells with *Staphylococcus aureus* [41] or when infecting macrophages with *Mycobacterium smegmatis* [42]. At a certain MOI, the percentage of infected cells stops increasing and it reaches a plateau [43]. The first MOI that allows reaching this plateau is defined as the optimal MOI (as many infected cells as possible). As described for *Yersinia* spp., cell viability should also be considered when defining MOI values. Therefore, a compromise must be made between an optimal MOI to obtain sufficiently high number of infected cells and one that will not compromise the viability of cells. Dependent on the research question, appropriate MOIs typically range from 5 to 100 (Table S1). Of note, even at an optimal MOI, not all cells will be infected; Jantsch et al. [44] reported that, upon infection of bone marrow derived DCs (BMDCs)—in which *Salmonella* spp. are not replicative—only 40% of the BMDCs were infected as determined by flow cytometry. The absence of replication in these cells makes omics studies more challenging given the limited amount of pathogenic material available. Interestingly, however, this model resembles the early time points of *Salmonella* spp. infection in the case of macrophages and epithelial cells when bacteria are not yet replicative. In another study performed in HeLa cells using the same *Salmonella* strain [30], the number of intracellular bacteria was found to differ among infected cells throughout time. Using mCherry-labeled bacteria, the number of intracellular replicative *Salmonella* bacteria was counted by fluorescence microscopy and it was found that 2 hpi, infected cells contained about 20 bacteria, whereas at 8 and 16 hpi this number ranged between 20 and 100. Overall, these examples clearly illustrate that not all cells become infected and, and when considering infected cells, the number of intracellular bacteria per cell can vary greatly.

Finally, the growth phase and composition of the media used during in vitro infections must be considered. For example, when studying *Salmonella* spp. invasion of epithelial cell lines, the late exponential phase was shown to be the phase during which *Salmonella* pathogenesis island-1 (SPI-1) is highly induced, and therefore the more suitable growth phase for infection [45]. Many studies make use of opsonizing antibodies or proteins of the complement system present in serum when phagocytic cells are used [46]. Opsonization is carried out to ensure bacterial internalization into phagocytic cells by phagocytosis, a key process for host defense during infection [47]. In case of *F. tularensis* infecting bone marrow derived macrophages, opsonized bacteria are more efficiently phagocytized than non-opsonized bacteria as shown by Geier et al. [48]. Moreover, when studying the Gram-positive pathogen *L. monocytogenes*, Kolb-Maurer et al. [49] showed that, upon replacing fetal calf serum (FCS) in the culture medium by human plasma, the bacteria were more actively taken up by the cells (Table S1). Additionally, when *Salmonella* was used to infect bone marrow derived macrophages [50], opsonization with mouse whole serum was performed prior to infection. It is also important to consider that the switch from bacterial to host cell growth media when performing the infection might introduce (substantial) changes in the gene expression profile of bacteria, necessitating the need for the inclusion of appropriate controls. Moreover, despite considering the intrinsic limitations when isolating samples for omics studies, it is important to consider a wide variety of experimental parameters when comparing the outcome of host pathogen interaction studies. We found data comparison to be complicated viewing the lack of essential information provided next to the diversity of technologies and instruments used and the wide variety of differing parameters among the experimental setups studied. More specifically, information on the MOI used, CFU recovered, bacterial growth phase, culture conditions of the host cells, infection conditions, time-points and sensitivity of instrument used to acquire the data is often lacking.

## 3. Transcriptome Analyses during Infection to Study Gene Expression Profiles of Host and Pathogen Simultaneously: RNA-Seq Supplanting Microarrays

Transcriptomics refers to the study of an organism’s transcriptome, which is the totality of its RNA transcripts. Hence, transcriptomics allows detection of the expressed gene pool. Transcriptomes have classically been studied using DNA microarrays [51]. Briefly, RNA is extracted from the cells, converted to cDNA via reverse transcription, fluorescently labeled by PCR amplification [52] and hybridized with immobilized gene-specific DNA probes. Upon hybridization, the fluorescence signals allow detecting the transcripts and quantification of their levels. Moreover, by using different fluorescent labels, multiple transcriptomes can be studied simultaneously [53]. Even though microarrays allowed the study of pathogen transcriptomes in infection models [54], with the advent of next-generation sequencing approaches enabling high-throughput sequencing, massively parallel cDNA sequencing or RNA-seq quickly took over. An extensive overview of RNA-seq approaches together with their technical advantages over microarray techniques has recently been reviewed [55,56]. 

RNA-seq holds some interesting advantages over microarrays that are important when studying host–pathogen interactions. More specifically, RNA-seq has a broader dynamic range than microarrays (10^5^ and 10^3^, respectively [57]), allowing for addressing larger fold-changes in gene expression. RNA-seq also enables detection of low abundance transcripts and allows discriminating between different transcript isoforms and identification of genetic variants. Important for host–pathogen studies and due to its high sensitivity [58], simultaneous gene expression profiling of both pathogen and host became possible. Moreover, as RNA-seq is sequencing-based and read mapping onto the respective genomes is performed in silico, cross-reactivity when studying different species, an important drawback of microarrays, does not any longer occur. More specifically, in recent years, several studies made use of dual RNA-seq, a term coined by Westermann et al. [56], when studying host–pathogen interactions using in vitro and ex vivo infection models (reviewed in [26]). When assuming 10 bacteria per infected cell, sequencing depth requirements to obtain correct reflections of both host and pathogen expression profiles were estimated to be 200 and 2000 million total reads for rRNA depleted and total RNA samples, respectively [56]. Of note, however, while these sequencing depths suffice to determine changes in gene expression, sequence coverage of the transcriptomes obtained this way is still too low to provide detailed information regarding transcript structure, delineation of transcript boundaries, etc. Using dual RNA-seq, some of the studied pathogens include *S. typhimurium* [38], *S. aureus* [59], uropathogenic *E. coli* (UPEC) [60], *Chlamydia trachomatis* [61], *Mycobacterium bovis* [62] and *Haemophilus influenzae* [63]. More recently, dual RNA-seq was also used to simultaneously study host and pathogen gene expression profiles using in vivo infection models (reviewed in [26]). Briefly, gene expression was analyzed in mice infected with *Pseudomonas aeruginosa*, after lung tissue resection [64]. In another study, Peyer’s patches from mice infected with *Yersinia pseudotuberculosis* were analyzed [65]. In both cases, infected tissues were isolated and homogenized prior to total RNA extraction for sequencing. Since multiple types of cells might be involved, this approach has also been generally referred to as multi RNA-seq [26].

While both in vitro [38] and in vivo approaches ideally require a priori isolation of infected cells and even though in vivo models of host–pathogen interactions represent optimal conditions to understand bacterial pathogenesis, the composite gene expression profiles originating from a variety of host cell types observed in the case of the latter complicate data interpretation even further and thus optimally require separation of the different types of infected cells. Moreover, increased heterogeneity in bacterial pathogen populations might add another level of complexity to multi RNA-seq studies, making it, for example, also necessary to differentiate between the intra- or extracellular origin of bacteria without a priori isolation of infected cells as for instance, *Salmonella* alternates between a proliferative intracellular stage and an extracellular disseminative stage [66]. Since efficient and cost-effective separation methods are still lacking, the use of multi RNA-seq to study host–pathogen interactions remains somewhat limited [26].

## 4. Proteomics of Bacterial Pathogens in the Context of the Host—Overcoming the Overwhelming Host Proteome Contribution

To obtain sufficient quantities of a pathogen’s proteome from the infected host, enrichment and isolation protocols have been devised. Overall, the main objective of these is to reduce the contribution of host proteins to the isolated bacterial proteome content, thereby increasing the overall sensitivity of mass spectrometry (MS) based detection of bacterial proteins. Intact bacteria can be isolated from lysates of infected host cells using different methods such as differential centrifugation, fluorescence activated cell sorting (FACS) based cell sorting and immune (magnetic) selection, all of which rely on intrinsic or acquired properties that enable their selection. Next to these strategies, various approaches have been reported that enable specific bacterial protein labeling with non-natural amino acids and subsequent enrichment of labeled proteins.

### 4.1. Physical Separation Based Approaches for Isolating Intact Bacterial Pathogens upon Infection

Centrifugation-based approaches make use of infected host cells that are lysed by specific detergents. Triton-X100 is typically used as it enables selective lysis of the membranes from eukaryotic cells, leaving the membranes of bacterial cells intact. Thus, detergent-based lysis of infected host cells allows the isolation of intact and viable bacteria upon centrifugation. Using differential centrifugation, Yanhua Liu and coworkers studied the proteome of intracellular *Salmonella* infected HeLa cells [67]. The authors recovered a large amount of bacteria (i.e., ~10^8^ bacteria). After MS analysis, 1675 *Salmonella* proteins and 251 host contaminants proteins (i.e., about 13% of all proteins identified) were identified on average. Differential centrifugation was also used to study the proteome of several bacterial pathogens in both in vitro and in vivo infection models. Examples include *Shigella* [68,69], *Listeria* [70,71,72], *Yersinia* [73] and *Brucella* [74] spp.

Upon internalization, certain pathogens end up in specific subcellular vacuoles; so-called bacteria containing vacuoles (BCVs) (i.e., *Salmonella*, *Legionella*, *Shigella*, *Mycobacterium*, *Yersinia* and *Francisella* spp.). Pathogens inside BCVs can manipulate host cells’ pathways by interacting with particular targets through secretion of effector molecules. For instance, *Salmonella* induces its own engulfment in epithelial cells and is found in a membrane-bound compartment, known as the *Salmonella*-containing vacuole (SCV). This compartment is specially adapted by the bacteria to allow its own survival and replication [75]. By means of subcellular fractionation of infected cells using sucrose-density centrifugation, SCVs can be fairly purely isolated with subsequent recovery of viable bacteria. In the work of Santos et al. [76], the authors report a recovery of about 30 million bacterial cells when infecting 60 million HeLa cells at an MOI of 100. Inclusion of an immunoprecipitation (IP) step, such as the SseF IP performed in [77] and targeting a *Salmonella* secreted effector residing in the SCV membrane, might also be considered as a bacterial enrichment method as it yields purer SCV fractions. However, viewing the losses associated with such a workflow, the input material required is high and the sample manipulations costly and labor intensive making it not very suitable for downstream proteomics analysis [77]. Finally, in the particular case of *Salmonella* spp., which can survive and replicate in the cytosol of infected epithelial cells [30,78], a drawback of this approach is that it only enriches the bacterial population inside SCVs, thus neglecting the (possible) contribution of cytosolic bacteria to the overall infection process.

Using FACS based isolation, Becker and coworkers purified GFP expressing *Salmonella* from ex vivo infected mouse spleen and caecum cells [79]. In their study, 370 *Salmonella* proteins could be identified from spleen isolates and 835 proteins from caecum. Nevertheless, host contaminants were frequently observed in both samples as from all identified proteins in the spleen and caecum samples 70% and 25% originated from host proteins, respectively.

Another strategy uses immune (magnetic) selection (I(M)S) of bacteria. Again, this strategy relies on the isolation of the microorganisms after selective lysis of host cells by detergents, followed by the use of specific antibodies raised against a particular microorganism [80]. These antibodies are obtained by immunization of animals and collection of antisera against accessible epitopes presented by the pathogen. Twine et al. used I(M)S [80] to isolate *Francisella* bacteria. In their study, spleen homogenates from infected mice were incubated with magnetic beads coupled with anti-*Francisella* rabbit sera, which allowed for the isolation of up to ~49% of all bacteria (80 million bacterial cells) present in the spleen homogenate [80]. 

Even though physical isolation-based methods, such as FACS and I(M)S, allow separating bacteria from the host upon infection, their application is biased and restricted by several aspects, such as the ability to genetically manipulate the microorganisms, the capacity to obtain specific antibodies and the amount of sample needed. For instance, genetic manipulation of the Gram-positive pathogen *S. aureus* to express fluorescent proteins cannot be easily achieved [81]. Moreover, the scarce availability of antibodies targeting specific pathogens limits the wide applicability of immunoselection. An alternative approach is based on labeling bacteria with gold (Au) or ferric oxide-core (FeOx) poly (vinyl alcohol) coated fluorescence-labeled nanoparticles (NP) [82]. These NPs are internalized by *S. aureus* prior to infection and allow their subsequent isolation by FACS or magnetic separation. It is noteworthy however, that NP engulfment has been shown to affect the normal growth of the bacteria as a longer lag phase was observed due to bacterial adaptation to the NPs. To further characterize this, the authors used quantitative proteome analysis to analyze the protein expression profile of bacteria upon incubation with or without NPs. Several proteins with different expression profiles were observed this way. Moreover, upregulation of proteins involved in the anti-oxidative stress response indicates that *S. aureus* is exposed to mild oxidative stress when grown in the presence of FeOx-NP. To conclude, all of these strategies are powerful methods and have been successfully applied to study host–pathogen interactions. Nevertheless, as reported in several studies, optimization is still needed to increase the purity of the bacterial proteins obtained.

### 4.2. Protein Labeling-Based Isolation of the Bacterial Proteome in Infection Models

**Figure 3:**
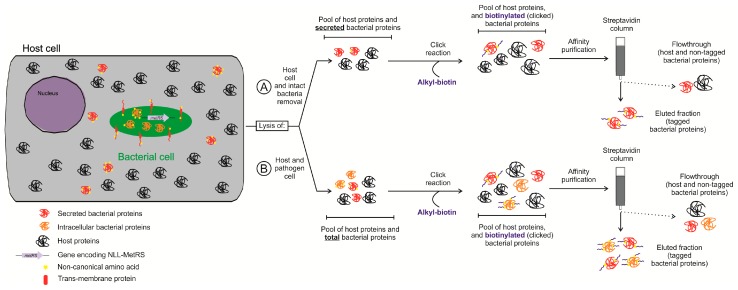
Bio-orthogonal labeling and purification of labeled bacterial (secreted) proteins from infected host cells. The scheme depicts the workflow for bio-orthogonal tagging of bacterial proteins with non-natural amino acids followed by click chemistry, and isolation of labeled pathogen proteins. Host cells are infected with bacteria, non-natural amino acids are added to the medium prior to infection (pre-labeled bacteria), or upon infection. Bacteria expressing the mutant methionyl-tRNA synthetase (NLL-MetRS), can incorporate non-natural Met analogous exclusively into bacterial proteins. Newly synthetized bacterial proteins will thus incorporate this non-natural Met. After infection, host cells are lysed either in (**A**) a selective manner keeping bacterial cells intact (e.g., to study the bacterial secretome) or (**B**) by complete lysis enabling to study the proteome of the pathogen and host simultaneously during infection in a more global manner. Particularly, in the case of (**A**), bacterial cells are typically removed by centrifugation before downstream processing of the obtained lysate. Subsequently, proteins are clicked using alkyl-biotin (blue). In this step, only proteins that have incorporated non-natural amino acid will be conjugated to biotin. Finally, a purification step using streptavidin affinity chromatography is performed. Here, non-tagged proteins do not bind the streptavidin resin and are thus easily removed, while the biotinylated (clicked) proteins can be recovered. Thus, bacterial (secreted) proteins are highly enriched in the sample and can be studied to understand changes in the secretome (**A**) or in the bacterial proteome (**B**) of a bacterial pathogen during infection.

In cellulo, labeling of bacterial proteomes is typically achieved using non-natural amino acids that are exclusively incorporated into the bacterial but not the eukaryotic proteome (Figure 3A). Given that selective labeling of bacterial proteins occurs before lysis of infected cells, such approaches do not require a priori physical separation of bacteria from host cells in contrast to the strategies described above [83,84]. For a comprehensive review of labeling approaches, we refer to Tirrell et al. [85]. Typically, functional groups of non-natural amino acids incorporated into bacterial proteins by the translation machinery enable specific derivatization of labeled proteins and their subsequent isolation [85]. Some of the non-natural amino acids used, such as the azide-bearing methionine surrogate azido homoalanine (AHA), can be loaded to methionyl-tRNA following wild-type aminoacyl-tRNA synthetase (aaRS) activation of AHA. Unlike AHA, however, trans-crotylglycine (Tcg) or azido norleucine (ANL), other methionine surrogates, are not activated (efficiently) by wild-type aminoacyl-tRNA synthetases and respectively require overexpression of methionyl-tRNA synthetase, or the expression of a mutant aaRS [85], enabling the (nearly) exclusive incorporation in bacterial proteins. One area of research where these approaches have been applied is the selective isolation and identification of the newly synthesized proteome [86,87]. More specifically, when AHA is added to the media for a (short) pulse, it will first be incorporated into newly synthesized proteins, an approach referred to as biorthogonal noncanonical amino acid tagging (BONCAT) [86]. Since AHA contains a biorthogonal azido group, a chemical group that does not interfere with native biochemical processes nor has any biological activity, upon extraction of the total proteome content, specific labeling of proteins containing AHA can be obtained by azide–alkyne cycloaddition also known as click chemistry [88]. Here, the alkyne group can be conjugated to different molecules, like fluorophores or biotin, which allows fluorescent based inspection (e.g., microscopy) or isolation by affinity purification respectively. Nevertheless, when studying host–pathogen interactions, labeling with AHA is not applicable because both the pathogen and host proteomes may incorporate this surrogate, as explained above.

A way to overcome this issue came with the discovery of variants of aaRS that can activate specific non-natural amino acids [89] since such aaRS mutants can be selectively expressed in bacteria but not in the host, which allows for selective labeling of bacterial proteomes in the context of the infected host [83,84]. More specifically, Ngo et al. [83] used an *E. coli* strain constitutively expressing mutant methionyl-tRNA synthetase (i.e., NLL-MetRS) [89] to infect macrophages in medium supplemented with ANL. After lysis of the infected cells, proteins were clicked with alkyne-functionalized biotin. Biotin-clicked bacterial proteins were subsequently enriched by streptavidin-aided affinity purification. In another study, Grammel and coworkers [84] ectopically expressed *E. coli* NLL-MetRS in *Salmonella*, which also enabled ANL incorporation into *Salmonella* proteins. Additionally, the authors used the alkynyl isostere of ANL, 2-amino-octynoic (AOA) instead of ANL [84], and showed that, in their experimental conditions, AOA resulted in a lower background signal typically resulting from non-specific cross-reactions. In this study, different AOA pulses were used to study the alteration of the *Salmonella* proteome in infected macrophages. Despite the fact that 85% of all protein identifications originated from *Salmonella*, only 195 *Salmonella* proteins were identified [84]. Even though biorthogonal labelling represents an alternative approach to avoid physical isolation methods, its implementation has the limitation that only proteins containing methionines will be (partially) labelled. In the case of *Salmonella*, 96% of its proteome contains one or more Met residues in addition to the N-terminal initiator Met residue. Moreover, the incorporation of non-natural amino acids into the proteins can have a metabolic effect as shown for the intracellular parasite *Toxoplasma gondii* [90], and therefore might affect the infection process.

## 5. Secretomics for Identifying Bacterial Extracellular Effectors Involved in Infection 

The term bacterial secretome was coined in studies of the eubacteria *Bacillus subtilis* by Tjalsma et al. in 2000 [91] and Antelmann et al. in 2001 [92]. It was initially used to refer to both the secreted proteins and the actual secretion machinery. However, the current paradigm in the field has shifted to define the secretome in a broader way as not only the subset of secreted proteins or so-called exoproteome [93], but also including bacterial proteins that interact with the extracellular environment such as the membrane-anchored adhesins which facilitate adherence to the surface or to host cells [94]. These proteins are part of the cell wall proteome and some of them have key roles in virulence, such as in the evasion of the host immune response [71].

Bacterial secretomes have been studied by different approaches including in silico identification of secretion signals, isolation of the secretomes of in vitro cultured microorganisms under different growth conditions and screening of proteins by phage display. In silico based approaches rely on algorithms to find consensus secretion signals characteristic of secreted proteins [95]. Secreted proteins are usually targeted to the secretory pathway by a signal either encoded in the N-terminal region of the protein or by the 5′ end of the mRNA sequence [96]. While in silico secretome predictions enable the rapid discovery of potential secreted proteins, empiric validation of candidates is required to show that these proteins indeed belong to the secretome. Such validation is not always straightforward, amongst others because proteins predicted to be secreted might be poorly expressed. Furthermore, this approach relies on proper genome annotation, which is not always available. In addition, in vitro techniques typically identify secreted proteins in culture supernatants after physical separation from the bacteria [97,98,99,100,101,102]. Particularly, this approach has been used to study secretomes of several species of the Gram-positive human pathogen *Streptococcus* under different growth conditions [103,104]. This way, more than 100 secreted proteins could be identified in each case. However, in such studies, bacteria are grown in conditions that only mimic the host environment. Hence, the actual set of proteins secreted upon interaction with the host might be different from the protein set obtained in vitro. Phage display is based on the generation of a library containing up to 10^12^ phage particles displaying different bacterial protein(s) (parts) [105]. This strategy for studying the secretome can be used when proteins interacting with host cells, the extracellular matrix, serum proteins such as antibodies and the complement system are used as baits to fish out those bacterial proteins that are potentially secreted or membrane associated [106]. Therefore, the use of phage display for the identification of secretome proteins is limited to those proteins interacting with host surfaces (reviewed by Gagic et al. [105]), thus missing the intracellular host targets of secreted proteins.

Recently, however, enrichment of bacterial secretomes using in vitro infection models was reported [107,108]. Both studies used biorthogonal labeling of bacterial proteins with ANL to isolate the secretome (Figure 3B). Mahdavi and coworkers [107] studied the secretome of *Yersinia enterocolitica* infected HeLa cells. In this work, effectors secreted by extracellular or intracellular *Yersinia* were identified. At the early stage of infection, *Yersinia* is extracellular and uses a type III secretion system (T3SS) to inject effectors into the host. Interestingly, infection can be detected due to the changed morphology of HeLa cells after injection of the effectors. In their experimental setup, *Yersinia* constitutively expressing NLL-MetRS was used to infect HeLa cells in media supplemented with ANL. Upon selective lysis of the host and removal of intact bacteria, effectors injected in host cells were enriched and subsequently identified by mass spectrometry. A similar strategy, but now including a step to eliminate extracellular bacteria by a gentamicin protection assay, was performed in [29]. This way, upon lysis of the host cells, protein secreted by intracellular bacteria could be identified. The outcome of these two strategies, was the identification of two different groups of secreted proteins; namely eight T3SS-effectors (three secreted by extracellular *Yersinia* and five by intracellular *Yersinia*) and five secreted effectors not secreted by T3SS as demonstrated when making use of a mutant strain deficient in type III secretion. Finally, pulses with ANL allowed for a time-lapse study of the secreted effectors during the process of *Yersinia* infection and the order of the different injected proteins could be established. Another study carried out by Chande and coworkers [108] also used ANL incorporation into mycobacterial proteins to identify secreted effectors by different virulent *Mycobacterium* strains infecting THP-1 monocytes at different time points. Here, a *Mycobacterium* codon-adapted NLL-MetRS was used and the bacteria labeled 12 h prior to infection. Presumably, pre-labeling is required due to the slow growth rate of *Mycobacterium* [109]. By using label-free proteomics, the authors could identify 15 to 30 secreted proteins in three different strains, and quantified differences in their abundance at different time points. Of note, the time points performed in this study differed 12 h in time, while the time points of *Yersinia* study described above were collected 30 min apart [107], again highly likely in line with the different growth rates exhibited by these pathogens.

## 6. Ribosome Profiling as the Next Big Step of Future Endeavors for Studying Bacterial Translatomes upon Host Cell Infection?

Despite the recent advances made in studying bacterial protein synthesis by means of proteomics, both the difficulty of distinguishing newly translated and nascent proteins from the pre-existing protein pools (e.g., when identifying newly translated bacterial proteins upon infection) next to the increasing capabilities of NGS in terms of throughput and sensitivity, spurred an awareness to start analyzing bacterial protein translation at the level of the transcriptome/genome.

Only recently, ribosome profiling (or Ribo-seq) revolutionized the study of translation by deep sequencing of ribosome-protected mRNA fragments [110], thereby expanding our current understanding of the genomic architecture and coding potential for a wide variety of organisms to an unprecedented extent [111]. Moreover, recent ribosome profiling studies performed by us and others enabled translatome studies of bacterial cells, not only revealing translation of numerous previously unidentified (small) open reading frames, so-called pseudogenes and translation from numerous alternative translation initiation events including near-cognate start sites [112,113,114,115], but also empowering differential gene expression studies under infection relevant conditions [116]. 

Unlike the multitude of viral translation studies reported in the context of infection [117], and likely complicated due to the significant differences between bacterial and eukaryotic translation, to date, no bacterial translatome-oriented studies in the context of infection have been reported. Interestingly, however, unlike strategies relying on physical methods to separate cell types and cell enrichment strategies, genetically encoded systems were reported studying mRNA translation in different cell (sub-) populations [118,119]. More specifically, by expressing an epitope tagged ribosomal protein in a cell-dependent manner or under a tissue-specific promotor, translating ribosome affinity purification (TRAP) and RiboTag approaches enabled tissue or cell-type specific profiling of translatomes by the selective recovery (i.e., immunopurification) of tagged polysome-associated mRNAs from cells expressing a tagged ribosomal subunit [120,121].

In principle, similar strategies making use of ribosome tagged bacterial strains [122] would permit selective isolation of bacterial ribosomes when studying the bacterial translatome in a host cell specific context. Moreover, as in the case of the multi RNA-seq strategies described above, it is still highly preferable to enrich for the desired subset of bacterial transcripts, especially in the context of newly translated proteins. Again, in silico discrimination between host and pathogen can simply be performed by mapping the data onto respective reference genomes.

## 7. Conclusions

The implementation of proteogenomics approaches for the study of host pathogen interactions encounters its major limitation in the discriminative power required to study pathogen and host simultaneously throughout the infection process. To try to overcome these limitations, several approaches in the fields of transcriptomics and proteomics have recently emerged, permitting host as well as pathogen focused proteome and transcriptome studies. Importantly, since for most bacterial pathogens, the complete repertoire of effectors employed to establish a successful infection remains unknown, by performing integrative analysis of gene and protein expression data, a more qualitative and refined set of true protein coding potential of the pathogen in its host context can be envisaged. Furthermore, by providing an unprecedented understanding of interactions between pathogen and host, the identification of potentially new pathogen virulence factors will be facilitated.

## Supplementary Materials

The following material is available online www.mdpi.com/2227-7382/5/4/26/s1, Table S1: Models used for studying host-pathogen interactions.
