# Supplementary material for: Proteogenomics in Aid of Host–Pathogen Interaction Studies: A Bacterial Perspective
Source: Proteomes. 2017 Oct 11;5(4):26. doi: 10.3390/proteomes5040026 (PMC5748561; doi:10.3390/proteomes5040026)
Supplement: Supplementary file 1 [file proteomes-05-00026-s001.pdf]

Table S1| Models used for studying host-pathogen interactions.

Bacterial Pathogen	Host Model(1)			Experimental Approach(2)	MOI(3)	Infection Efficiency(4)		Reference
	Cells	Type of Cell	Starting Amount (cells/well)			Infected Cells	Intracellular Bacteria (*)	
Salmonella Typhimurium	BMDC	Primary	ND	FACS<br>CFU	ND	40 %, 2 hpi	~10^4 CFU, 1 hpi	[1]
	RAW264.7	Macrophage-like				ND	~10^5 CFU, 1 hpi	
	HeLa	Epithelial	ND	IF	50	ND	5±4 /cell(**), 2 hpi	[2]
	RAW264.7	Macrophage-like	5 × 10^5<br>(24 h before infection)	CFU<br>IF	100	ND	~10-fold(**) increase, 8 hpi	[3]
	HeLa	Epithelial	5 × 10^5<br>(24 h before infection)			> 90%, 2 h pi	~10/cell(**), 8 hpi<br>~10-fold increase(**), 8 hpi	
	Swiss 3T3	Fibroblast	5 × 10^5<br>(24 h before infection)			ND	~10-fold increase(**), 8 hpi	
	HeLa	Epithelial	8 × 10^4<br>(24 h before infection)	IF<br>CFU	10	81 ± 5 %, 16 hpi	~2 x10^5 CFU, 14 h pi	[4]
	COS-7	Fibroblast	ND	IF	50	> 80 %, 16 hpi	ND	
	HeLa-S3	Epithelial	2 × 10^5<br>(48 h before infection)	FACS<br>CFU	5	ND	~10/cell, 4 h pi<br>~75/cell, 24 h pi	[5]
					10	< 25 %, 4 hpi	ND	
					100	> 75 %, 4 hpi	ND	
	HeLa	Epithelial	ND	CFU	100	ND	~3 fold increase 6 h pi	[6]
	RAW264.7	Macrophage-like			10		~20 fold increase 21.5 h pi	
Yersinia spp.	BMDC	Primary	3 × 10^3	FACS<br>CFU	20	65 %, 4 hpi *Y. enterocolitica*	550/100 cells, 4 hpi<br>124/100 cells, 1 dpi<br>27/100 cells, 3 dpi	[7]
					50	> 95 %, 4 hpi *Y. enterocolitica*	ND	
	BMM and PEM	Primary	3 – 5 × 10^6	FACS	20	~40 % *Y. pestis*<br>~70 % *Y. pseudotuberculosis*<br>3 hpi	ND	[8]
	HeLa	Epithelial	2 × 10^4<br>(48 h before infection)	CFU	10	~40 % *Y. pestis*, 2 hpi	ND	[9]
	hMM	Primary	ND	IF	2	~50 % *Y. pestis*, 2 hpi	~4/cell, 2 hpi	[10]
	BMM	Primary	1 × 10^5	CFU	50	ND	10^3 CFU, 1 hpi	[11]
	J774.1	Macrophages	2 × 10^5	CFU	50	ND	~10^{10} CFU/mL(***) 24 hpi	[12]

	THP-1	Monocytes	2×10^5			ND	~10^9 CFU/mL(***)24 hpi	
	J774.1	Macrophages	2×10^5	CFU	50	ND	~10^8 CFU/mL(***) 24 hpi	[13]
	THP-1	Monocytes	2×10^5				~10^8 CFU/mL(***) 24 hpi	
	BMM	Primary	2×10^5				~10^8 CFU/mL(***) 24 hpi	
	hMM	Primary	ND				~10^9 CFU/mL(***) 24 hpi	
	hNeu	Primary	ND				~10^7 CFU/mL(***) 2 hpi	
	BMM	Primary	ND	IF	50	> 10 %, 4 hpi<br>> 20 %, 24 hpi	~1/cell, 24 h pi	[10]
	hMM	Primary	ND			> 15 %, 4 hpi<br>> 30 %, 24 hpi	~2/cell(**), 24 h pi	
Listeria monocytogenes	hNeu	Primary	ND	IF	1	> 80 %, 0.5 hpi	4.24±0.44/cell(**), 0.5 hpi	[14]
					10	> 90 %, 0.5 hpi	5.38 ± 0.77/cell(**), 5 hpi	
	BMM	Primary	2×10^5	CFU	20	ND	20/cell, 1 hpi	[15]
	Caco-2	Epithelial	ND	FACS	50	30 %, 3 hpi	ND	[16]
	hDC	Primary	5×10^5	EM	50	> 80 %, 1.5 hpi	10 – 35/cell, 1.5 hpi	[17]
	hGran	Primary	ND	FACS<br>CFU	20	> 20 %, 1 hpi	214±188/100 cells, 1 hpi (female donors)<br>80±51/100 cells, 1 hpi (male donors)	[18]
	hMono	Primary	ND			> 95 %, 1 hpi	344±209/100 cells, 1 hpi (female donors)<br>113±42/100 cells, 1 hpi (male donors)	

(*) Number of bacteria per cell, CFU and/or increase in CFU (post infection) observed
(**) Microscopy based
(***) Total volume of the lysate unspecified

(1) **BMDCs**: Bone Marrow derived Dendritic Cells; **BMMs**; Bone Marrow derived Macrophages, **PEMs**: Peritoneal Macrophages; **h:** human; **MM**: Monocyte derived Macrophage; **Neu**: Neutrophils; **Mono**: Monocytes; **Gran**: Granulocytes; **M**: Mouse; **ND**: Not described.
(2) **CFU**: Colony Forming Units; **IF**: Immunofluorescence; **FACS**: Fluorescent Assisted Cell Sorting; **EM**: Electron Microscopy.
(3) **MOI**: Multiplicity Of Infection; **ND**: Not described.
(4) **hpi**: hours post-infection; **dpi**: days post-infection; **ND**: Not described

References

1. Jantsch, J., et al., *Intracellular activities of Salmonella enterica in murine dendritic cells.* Cell Microbiol, 2003. **5**(12): p. 933-45.
2. Malik-Kale, P., S. Winfree, and O. Steele-Mortimer, *The bimodal lifestyle of intracellular Salmonella in epithelial cells: replication in the cytosol obscures defects in vacuolar replication.* PLoS One, 2012. **7**(6): p. e38732.

3. Beuzon, C.R., S.P. Salcedo, and D.W. Holden, *Growth and killing of a Salmonella enterica serovar Typhimurium sifA mutant strain in the cytosol of different host cell lines.* Microbiology, 2002. **148**(Pt 9): p. 2705-15.
4. Abrahams, G.L., P. Muller, and M. Hensel, *Functional dissection of SseF, a type III effector protein involved in positioning the salmonella-containing vacuole.* Traffic, 2006. **7**(8): p. 950-65.
5. Westermann, A.J., et al., *Dual RNA-seq unveils noncoding RNA functions in host–pathogen interactions.* Nature, 2016. **529**(7587): p. 496-501.
6. Brumell, J.H., et al., *SifA permits survival and replication of Salmonella typhimurium in murine macrophages.* Cell Microbiol, 2001. **3**(2): p. 75-84.
7. Schoppet, M., A. Bubert, and H.I. Huppertz, *Dendritic cell function is perturbed by Yersinia enterocolitica infection in vitro.* Clin Exp Immunol, 2000. **122**(3): p. 316-23.
8. Bi, Y., et al., *Yersinia pestis versus Yersinia pseudotuberculosis: effects on host macrophages.* Scand J Immunol, 2012. **76**(6): p. 541-51.
9. Cowan, C., et al., *Invasion of Epithelial Cells by Yersinia pestis: Evidence for a Y. pestis-Specific Invasin.* Infection and Immunity, 2000. **68**(8): p. 4523-4530.
10. Ireland, R., et al., *Effective, broad spectrum control of virulent bacterial infections using cationic DNA liposome complexes combined with bacterial antigens.* PLoS Pathog, 2010. **6**(5): p. e1000921.
11. Geier, H. and J. Celli, *Phagocytic Receptors Dictate Phagosomal Escape and Intracellular Proliferation of Francisella tularensis.* Infection and Immunity, 2011. **79**(6): p. 2204-2214.
12. McRae, S., et al., *Inhibition of AcpA phosphatase activity with ascorbate attenuates Francisella tularensis intramacrophage survival.* J Biol Chem, 2010. **285**(8): p. 5171-7.
13. Mohapatra, N.P., et al., *Type A Francisella tularensis acid phosphatases contribute to pathogenesis.* PLoS One, 2013. **8**(2): p. e56834.
14. Arnett, E., et al., *The pore-forming toxin listeriolysin O is degraded by neutrophil metalloproteinase-8 and fails to mediate Listeria monocytogenes intracellular survival in neutrophils.* J Immunol, 2014. **192**(1): p. 234-44.
15. de Chastellier, C. and P. Berche, *Fate of Listeria monocytogenes in murine macrophages: evidence for simultaneous killing and survival of intracellular bacteria.* Infect Immun, 1994. **62**(2): p. 543-53.
16. Balestrino, D., et al., *Single-Cell Techniques Using Chromosomally Tagged Fluorescent Bacteria To Study Listeria monocytogenes Infection Processes.* Applied and Environmental Microbiology, 2010. **76**(11): p. 3625-3636.
17. Kolb-Maurer, A., et al., *Listeria monocytogenes-infected human dendritic cells: uptake and host cell response.* Infect Immun, 2000. **68**(6): p. 3680-8.
18. Raybourne, R.B., et al., *Uptake and killing of Listeria monocytogenes by normal human peripheral blood granulocytes and monocytes as measured by flow cytometry and cell sorting.* FEMS Immunol Med Microbiol, 2001. **31**(3): p. 219-25.
